# Intercostal Catheters for Postoperative Pain Management in VATS Reduce Opioid Consumption

**DOI:** 10.3390/jcm10020372

**Published:** 2021-01-19

**Authors:** Florian Ponholzer, Caecilia Ng, Herbert Maier, Hannes Dejaco, Andreas Schlager, Paolo Lucciarini, Dietmar Öfner, Florian Augustin

**Affiliations:** 1 Center of Operative Medicine, Department of Visceral, Transplant and Thoracic Surgery, Medical University of Innsbruck, 6020 Innsbruck, Austria; florian.ponholzer@tirol-kliniken.at (F.P.); caecilia.augustin@tirol-kliniken.at (C.N.); herbert.maier@tirol-kliniken.at (H.M.); paolo.lucciarini@tirol-kliniken.at (P.L.); chirurgie@i-med.ac.at (D.Ö.); 2 Department of Anaesthesiology and Critical Care, Medical University of Innsbruck, 6020 Innsbruck, Austria; hannes.dejaco@tirol-kliniken.at (H.D.); andreas.schlager@i-med.ac.at (A.S.)

**Keywords:** minimally invasive, VATS, pain, postoperative pain control, thoracic surgery, lung cancer, intercostal catheter, opioid, regional anaesthesia

## Abstract

Background: Postoperative pain after video-assisted thoracoscopic surgery (VATS) affects patients’ recovery, postoperative complications, and length of stay (LOS). Despite its relevance, there are no guidelines on optimal perioperative pain management. This study aims to analyse the effects of an additional intercostal catheter (ICC) in comparison to a single shot intraoperative intercostal nerve block (SSINB). Methods: All patients receiving an anatomic VATS resection between June 2019 and May 2020 were analysed retrospectively. The ICC cohort included 51 patients, the SSINB cohort included 44 patients. Results: There was no difference in age, gender, comorbidities, or duration of surgery between cohorts. Pain scores on the first postoperative day, after chest drain removal, and highest pain score measured did not differ between groups. The overall amount of opioids (morphine equivalent: 3.034 mg vs. 7.727 mg; *p* = 0.002) as well as the duration of opioid usage (0.59 days vs. 1.25 days; *p* = 0.005) was significantly less in the ICC cohort. There was no difference in chest drain duration, postoperative complications, and postoperative LOS. Conclusions: Pain management with ICC reduces the amount of opioids and number of days with opioids patients require to achieve sufficient analgesia. In conclusion, ICC is an effective regional anaesthesia tool in postoperative pain management in minimally invasive thoracic surgery.

## 1. Introduction

Comparing post-operative pain regimens for video-assisted thoracoscopic surgery (VATS) across the literature, a wide variety and combinations of different drugs and techniques is found to be used without a universal standard. Currently, single shot intraoperative intercostal nerve block (SSINB)—also referred to as paravertebral block (PVB) or thoracic epidural analgesia (TEA)—is considered the gold standard for pain management after thoracotomy; however, guidelines are lacking for a VATS approach [1,2,3,4].

TEA catheter placement is an effective method for postoperative pain control, but also carries specific risks (e.g., epidural hematoma or spinal cord injury) and is also time consuming, not only because of the procedure itself but also because of the management of frequently occurring hypotension, which develops in 36–75% of patients [5,6,7]. Additionally, reported failure rates of placed catheters range from 5.6% to 30% [8,9]. An optional technique of pain management for VATS patients is the placement of an intercostal catheter (ICC). Recent studies of ICC seem to provide inconclusive results across institutions. This may be owed to most of the studies being of retrospective character or having a small sample size [10,11].

In the presence of Enhanced Recovery After Surgery^®^ (ERAS) protocols to improve outcome after surgery, thoracic surgery clearly needed to improve postoperative pain. Minimally invasive thoracic surgery significantly reduced postoperative pain in comparison to anterolateral thoracotomy in a controlled randomized trial [12]. However, a VATS approach is not free of pain. To further improve, there is a need to establish guidelines for reliable and effective pain management after VATS. This might not only impact patient satisfaction, but also help compete against the rising budgetary pressure for health care providers experienced worldwide, as the needed rehabilitation phase and rate of postoperative chronic pain might be decreased [13,14,15]. Moreover, in the current wave of the opioid epidemic, it is especially important to also focus on the role of opioids in postoperative pain management and possibilities to reduce their usage [16,17].

The aim of our study was to analyse the effect of an ICC in addition to PVB on post-operative pain, amount of opioid usage, and length of stay after surgery.

## 2. Experimental Section

### 2.1. Patient Selection

All patients from June 2019 to May 2020 receiving an anatomical VATS resection (lobectomy and segmentectomy) for primary lung cancer at our surgical institution were analysed retrospectively. Exclusion criteria were contraindications for opioid usage (one patient). Permission for analysis was granted by the local ethics committee (registration number: UN4424, 303/4.10).

A total of 95 consecutive patients were included in our database for further analysis. ICC placement was introduced in September 2019. Placement of an ICC was only attempted if the patient gave informed consent (one patient refused ICC placement). Four patients with primary non-function of the ICC (i.e., intraoperatively detected malposition of the catheter) were analysed in the PVB cohort. Furthermore, ICC placement intraoperatively was left at the discretion of the surgeon (15 patients after September 2019 without ICC).

### 2.2. Data Collection

Patients’ data were collected in a prospectively maintained database. Recorded data included patients’ age, gender, comorbidities (coronary artery disease, chronic obstructive pulmonary disease, and diabetes mellitus), type of operation, length of operation, length of stay, placement of an ICC, duration until chest drain removal, postoperative opioid usage, postoperative complications, and pain scores.

### 2.3. Definitions

#### 2.3.1. Study Endpoints

Primary study endpoint was defined as opioid consumption. Secondary study endpoints were defined as amount of opioid usage, duration of opioid usage, length of operation, chest drain duration, length of stay (LOS), and postoperative complications. Patient characteristics were also analysed.

#### 2.3.2. Surgical Technique

VATS resections follow a standardized procedure with a three-port approach using the Copenhagen technique and have been described elsewhere [18]. One camera incision is made in the seventh intercostal space, an auxiliary port incision is made in the eighth intercostal space, and a utility port incision is made in the fourth or fifth intercostal space. Thoracic drain was inserted in the camera incision at the end of the procedure.

#### 2.3.3. Analgesic Technique

All patients received general anaesthesia based on an in-house standard, which consists of either a combination of propofol and remifentanil (total intravenous anaesthesia, TIVA) or balanced anaesthesia using sevoflurane and remifentanil, depending on patient comorbidities. At the end of the operation, patients received 1 to 2 g of metamizole, 0.5 to 1 g of paracetamol, and 4.5 to 7.5 mg of piritramide for pain control, all depending on each patient’s weight. All patients received single-shot intercostal injections of bupivacaine 2.5 mg/mL under visual control at the end of the procedure covering the intercostal nerves III–IX. Postoperative pain management consisted of paracetamol and metamizole on a fixed schedule. Piritramide was only administered on request at numeric rating scale (NRS) > 5, administration of rescue medication was documented in the patient chart (time and amount). In case of repeated opioid request, other opiates might have been prescribed according to the preference of the surgeon. For statistical analysis, all prescribed opioids were converted to their morphine equivalent. Duration of opioid usage was defined as the time from surgery until the time of last opioid request during hospital stay.

**Figure 1:**
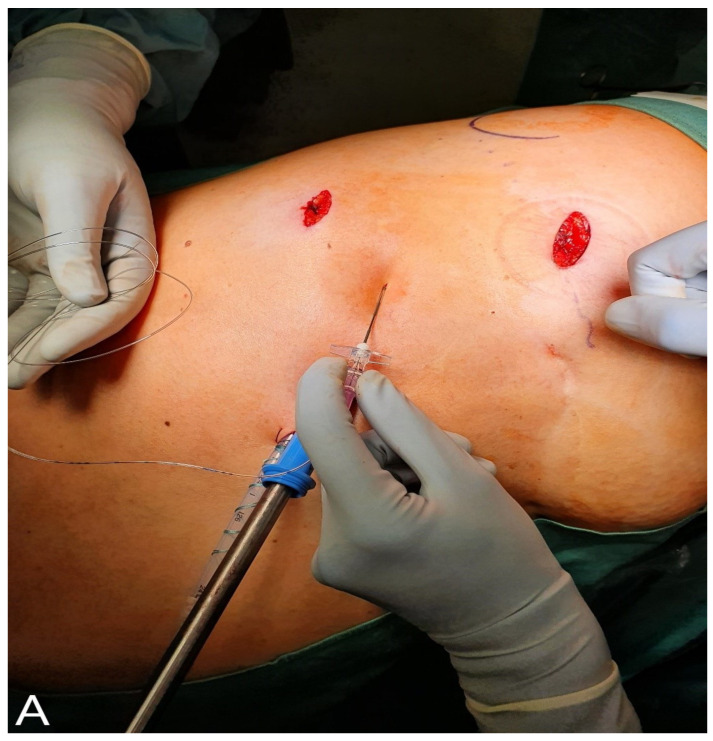

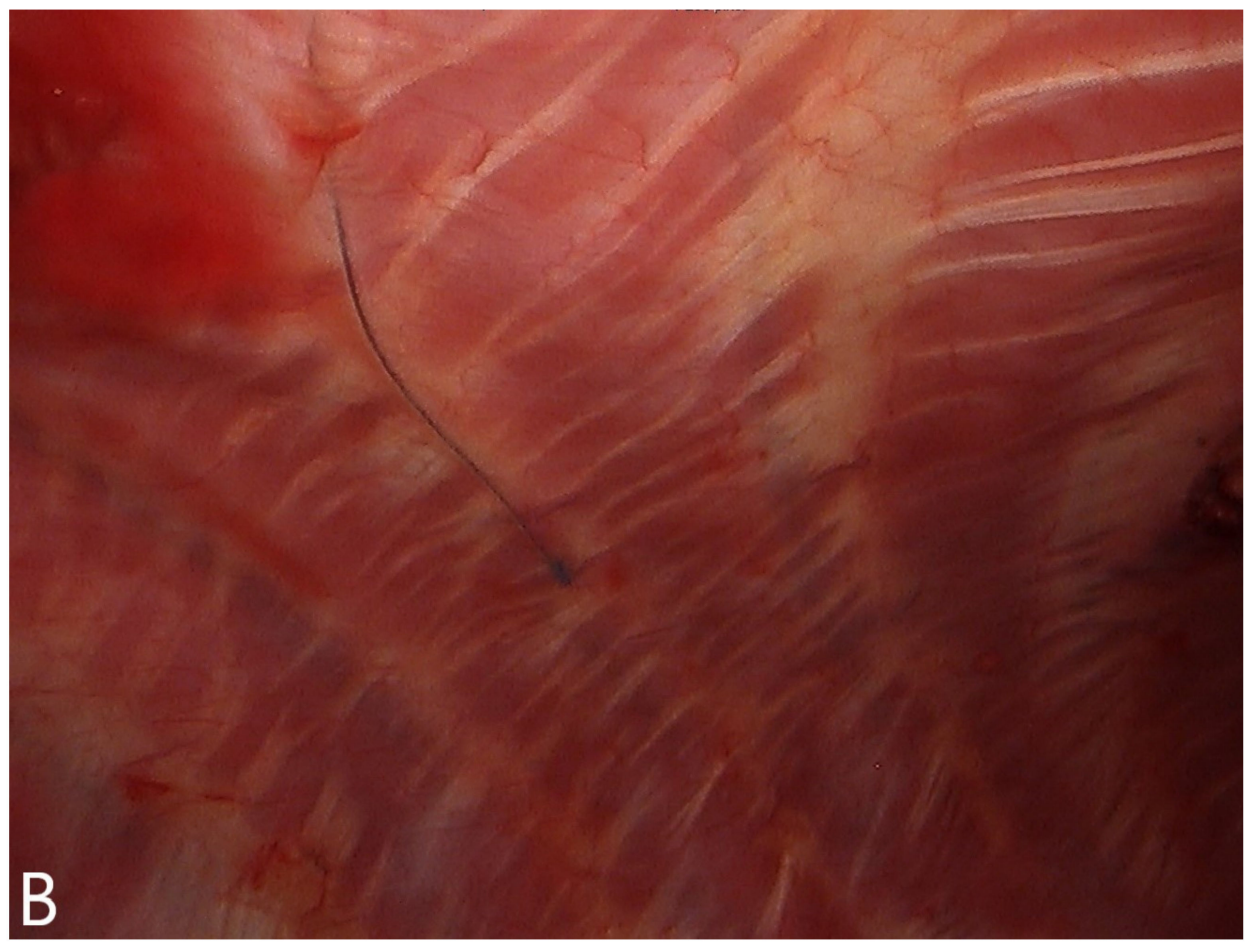
(**A**) View of the intercostal catheter (ICC) after insertion using a 16 Gauge Tuohy needle and a standard peridural catheter. (**B**). Visual control of the ICC. The ICC is placed in the same intercostal space as the chest drain. In the projection of the ICC, the corresponding intercostal nerve and vessels can be seen.

At the end of surgery ICCs were placed following a standardized technique. We used a regular 16G Tuohy needle and a catheter also used for peridural anaesthesia. The ICC was inserted in the same intercostal space as the chest drain, as can be seen in Figure 1A,B. Through the ICC, 2 mg/mL of ropivacaine was applied at a fixed rate of 6 mL/h with the same pumps as for epidural administration. ICCs were removed at the time of chest drain removal, or on pod 3 if the chest drain was kept in place because of an air leak.

#### 2.3.4. Pain Scoring

Pain Scoring by NRS was performed by staff nurses at least three times daily and was guided by the same cutpoints as described by Serlin et al. [19] with 0 meaning no pain, 1–4 indicating mild pain, 5–6 indicating moderate pain, and 7–10 indicating severe pain. Pain scores were documented in the hospital information system.

#### 2.3.5. Postoperative Complications

Postoperative complications were graded according to the Clavien–Dindo classification by Dindo et al. [20] and also split in pulmonary and non-pulmonary complications.

#### 2.3.6. Statistical Analysis

A *t*-test was performed for analysing means and Pearson’s chi-squared test was used to calculate correlations between categorical variables. A Kolmogorov–Smirnov test was used for analysing distribution; Mann–Whitney U test was used for comparing medians. Statistical significance was assumed for a *p*-value < 0.05. SPSS 26 (IBM Corp., Armonk, NY, USA) was used to perform statistical analysis.

## 3. Results

A total of 95 consecutive patients were analysed, with 51 (53.68%) being in the ICC and 44 (46.32%) in the SSINB cohort. Patients’ characteristics are shown in Table 1.

All patients received a primary VATS anatomic resection. There was no difference in age, gender, or number of drains placed between the two groups (*p* = 0.777/1.000/1.000, respectively). The median length of the operation was 2.5 min longer in the ICC group (ICC vs. SSINB: 145.00 vs. 142.50 min, respectively, *p* = 0.474 using the exact sampling distribution of U), which was attributed to the placement of the ICC; the difference was not significant. Mean length of operation also did not differ (153.84 vs. 144.27 min, respectively, *p* = 0.153). There was no injury to the intercostal vessels or nerve during the placement of the ICC.

Median chest drain duration and median postoperative LOS did not differ between groups (3.00 vs. 3.00 days, *p* = 0.766 using the exact sampling distribution of U; 6.00 vs. 6.00 days, *p* = 0.172 using the exact sampling distribution of U). There was no difference in the amount or type of postoperative complications (overall: *p* = 0.479; pulmonary complication vs. non-pulmonary complication: *p* = 0.675).

### Opioid Usage

**Figure 2:**
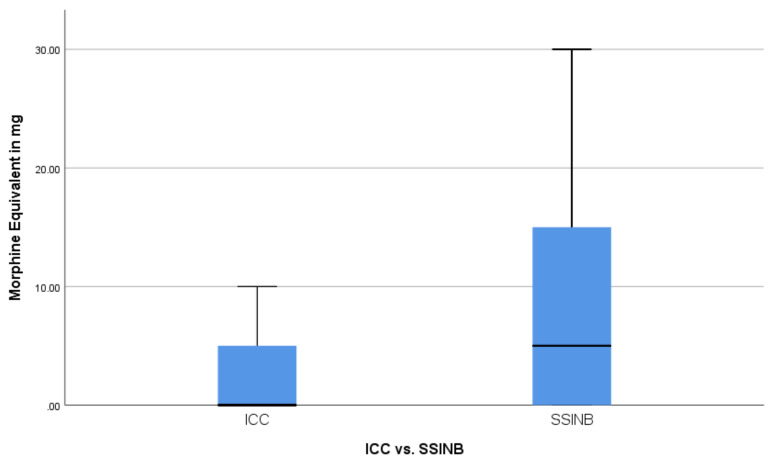
Distribution of morphine equivalent consumption between the ICC (intercostal catheter) and SSINB (single shot intercostal nerve block) group.

**Figure 3:**
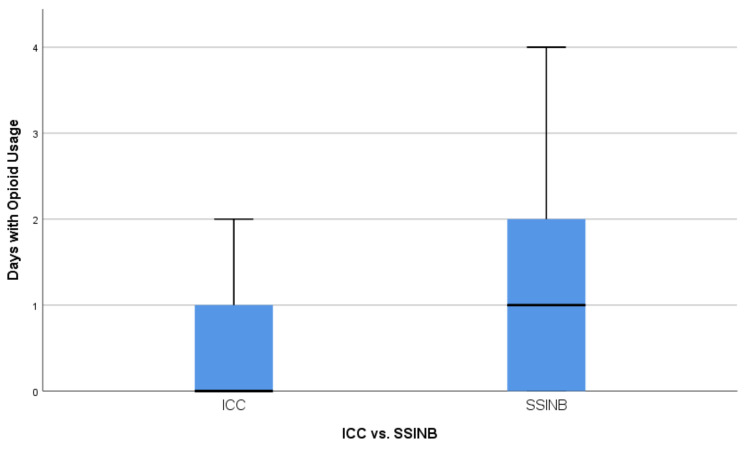
Distribution of days with opioid usage between the ICC (intercostal catheter) and SSINB (single shot intercostal nerve block) group.

To avoid statistical misinterpretation, both median and mean values were compared for opioid usage between the groups. The median total opioid usage was 0.000 mg morphine equivalent in the ICC cohort and 5.000 mg in the SSINB cohort (*p* = 0.012 using the exact sampling distribution of U, *r* = 0.256), as can be seen in Figure 2. The ICC cohort showed a significantly lower mean total opioid usage (morphine equivalent: 3.034mg vs. 7.727mg; *p* = 0.002). The median duration of opioid usage was 0 days in the ICC cohort and 1 day in the SSINB cohort (*p* = 0.014 using the exact sampling distribution of U, *r* = 0.251) (Figure 3). The mean duration of opioid usage was significantly lower in the ICC cohort (0.59 days vs. 1.25 days; *p* = 0.005).

The number of patients needing opioids was lower in the ICC cohort (43.1% vs. 59.1%, *p* = 0.151), but did not prove to be statistically significant. However, only 11.8% in the ICC group needed opioids for longer than one day, in contrast to 38.6% in the SSINB group (*p* = 0.010).

## 4. Discussion

Up to 40% of patients suffer from persistent pain as a result of acute postoperative pain in thoracotomy patients [1,21]. The introduction of minimally invasive surgery has significantly improved the outcome of patients undergoing lung surgery in contrast to thoracotomy, with reduced postoperative pain, improvement of respiratory function and quality of life, and shorter length of stay [12,22]. Despite this evidence, a VATS approach is not pain free. Sufficient pain control in the postoperative period is known to decrease postoperative morbidity and mortality and reduces the rate of chronic postsurgical pain after thoracic procedures [1]. However, there is no evidence for an ideal pain management regimen after VATS resections, and a variety of different treatment algorithms have been described in the literature with or without the use of regional anaesthesia.

Driven by the Enhanced Recovery After Surgery^®^ concept, we wanted to reduce the amount of opioids by introducing regional anaesthesia, thereby also reducing the amount of associated complications such as nausea, emesis, or hypotension. As opioids act as respiratory depressant, a lower opioid usage might also mitigate the risk of developing postoperative atelectasis and possible pneumonia [23,24].

In the search for an ideal regional anaesthetic procedure, we were specifically looking for an easy-to-perform and time-saving procedure with a low risk for associated complications. While epidural anaesthesia achieves good pain control, it is time-consuming, difficult to perform, and has the risk of damaging the spinal cord and postoperative hypotension [5,6,7]. Therefore, the use of peridural analgesia in minimally invasive thoracic surgery remains a matter of debate [25].

We introduced the technique of ICC at our department in September 2019. The catheter can easily be placed at the end of the procedure. It can be performed under visual control, thereby reducing the risk of direct damage to the intercostal vessels or nerve. Local anaesthetic is directly administered to the site of maximum pain in the postoperative period, which in most cases is the area of the chest drain [26]. According to our data, placing the catheter takes a median of approximately 2.5 min and does not result in ICC-related haematoma or nerve damage.

In our study, we were able to demonstrate a reduction of the total amount of postoperative opioids and the overall duration of opioid usage through placement of an ICC by 60.74% and 52.80%, respectively. Moreover, the rate of single-day opioid usage in contrast to multiple days was significantly less in the ICC group, pointing at better overall pain control with only little benefit experienced with rescue medication. This finding is in accordance with various ERAS protocols and pain management regimens by trying to limit the use of opioids and their potential side effects [27,28]. With regard to the ongoing opioid crisis, ICCs have been shown to be an appropriate adjuvant therapy for postoperative pain management [16,17]. Although our cohort consisted only of VATS resections, ICCs have also proven to be a feasible alternative to TEA in thoracotomy patients, as described by Luketich et al. [29], reducing the duration of supplemental opioid usage. In comparison to our described procedure, Luketich et al. [29] performed the catheter insertion by creating a tunnel over a minimum of two intercostal spaces above and below the thoracotomy, using a Stern clamp (Scanlon, St Paul, MN, USA) and pulling the catheter through. Further prospective investigations at our department might evaluate the combination of our ICC insertion procedure with the approach to cover more than one intercostal space in VATS and thoracotomy cohorts.

Possible confounders were ruled out, as we analysed a consecutive patient cohort without a selection bias, and statistical analysis showed no differences in comorbidities, age, or gender. Also, the time until removal of the thoracic drain did not differ between groups and therefore cannot explain the reduced duration of opioid usage.

Implementation of ICCs in our surgical standard proved to be rather frictionless, because it did not add significant delay to the operative time. Postoperative monitoring of the used pumps is performed by our in-house anaesthetists and simplified by using the same pump as for epidural administration, so there was no need for any additional investment/acquisition of medical devices.

Unfortunately, the improved pain management did not translate into reduced postoperative complications or reduced length of stay in this group with low overall morbidity of 25.3%. This might be explained by the still adequate pain control in the SSINB group using rescue medication. However, side effects of opioid usage are not routinely documented and due to the retrospective nature of the study are impossible to identify.

Our results suggest the additional use of regional anaesthesia through ICCs for optimizing postoperative recovery and pain management. Through the combination of ICC, intraoperative single-shot intercostal injections of bupivacaine, oral pain medication, and physical therapy, patients’ postoperative pathway can be optimized, resulting in better pain control, reduced breakthrough pain, presumably improved recovery and quality of life, and less opioid consumption.

### Limitations

This study was performed in a retrospective and non-randomized setting. The placement of ICCs at our department was started in September 2019 and thus the respective learning curve might have had an impact on the outcome. As the postoperative rate of complications and length of stay did not differ between groups, it is important to also focus on the patients’ quality of life and return to work after they are discharged from hospital. In regard to the surging budgetary pressure in the public health care sector and the rising number of resectable lung cancer diagnoses, it is important to prioritize this topic to reduce the strain on mentioned public health care providers [30,31]. Quality of life was not assessed in our study; however, this should be an integral part of any future prospective trials in the field of postoperative pain management.

## 5. Conclusions

As demonstrated in our study, through the standardized use of ICCs the postoperative need and duration for opioids can be minimized. ICCs represent an easy-to-perform procedure of adjuvant pain management for VATS anatomic lung resections. Further studies investigating combinations of various treatment modalities need to be performed in order to optimize postoperative pain management regimens and improve length of stay, return to daily routine, and rehabilitation.

## Figures and Tables

**Table 1:** Patient characteristics.

Factor	ICC, *n* = 51	SSINB, *n* = 44	*p*-Value
Age (years), median (range)	65 (28–83)	65 (37–80)	0.993
Gender (%)			1.000
Female	26 (51.0)	23 (52.3)	
Male	25 (49.0)	21 (47.7)	
Side (%)			1.000
Left lung	16 (31.4)	14 (31.8)	
Right Lung	35 (68.6)	30 (68.2)	
Lobe (%)			1.000
Upper Lobe	24 (47.1)	21 (47.7)	
Middle Lobe	5 (9.8)	4 (9.1)	
Lower Lobe	21 (41.2)	19 (43.2)	
Multilobar	1 (2.0)	0 (0.0)	
Comorbidities (%)			
Coronary Artery Disease	7 (13.7)	7 (15.9)	0.780
Chronic Obstructive	12 (23.5)	13 (29.5)	0.641
Pulmonary Disease			
Diabetes Mellitus	5 (9.8)	7 (15.9)	0.537
Postoperative Complications (%)			0.721
Clavien-Dindo I–II	8 (15.7)	9 (20.5)	
Clavien-Dindo III–IV	3 (5.9)	4 (9.1)	
No Complication	40 (78.4)	31 (70.5)	

Abbreviations: SSINB: Single Shot Intraoperative Intercostal Nerve Block; ICC: Intercostal Catheter.

## Data Availability

The data presented in this study are available on request from corresponding author. The data are not publicly available due to privacy reasons.

## References

[1] Goto T. (2018). What is the best pain control after thoracic surgery?. J. Thorac. Dis..

[2] Mercieri M., D’Andrilli A., Arcioni R. (2018). Improving postoperative pain management after video-assisted thoracic surgery lung resection contributes to enhanced recovery, but guidelines are still lacking. J. Thorac. Dis..

[3] Elmore B., Nguyen V., Blank R., Yount K., Lau C. (2015). Pain management following thoracic surgery. Thorac. Surg. Clin..

[4] Steinthorsdottir K.J., Wildgaard L., Hansen H.J., Petersen R.H., Wildgaard K. (2014). Regional analgesia for video-assisted thoracic surgery: A systematic review. Eur. J. Cardiothorac. Surg..

[5] Hansdottir V., Philip J., Olsen M.F., Eduard C., Houltz E., Ricksten S. (2006). Thoracic epidural versus intravenous patient-controlled analgesia after cardiac surgery: A randomized controlled trial on length of hospital stay and patient-perceived quality of recovery. Anesthesiology.

[6] Hewson D.W., Bedforth N.M., Hardman J.G. (2018). Spinal cord injury arising in anaesthesia practice. Anaesthesia.

[7] Ferré F., Martin C., Bosch L., Kurrek M., Lairez O., Minville V. (2020). Control of spinal anesthesia-induced hypotension in adults. Local Reg. Anesth..

[8] Gleicher Y., Singer O., Choi S., McHardy P. (2017). Thoracic epidural catheter placement in a preoperative block area improves operating room efficiency and decreases epidural failure rate. Reg. Anesth. Pain Med..

[9] McLeod G., Davies H., Munnoch N., Bannister J., MacRae W. (2001). Postoperative pain relief using thoracic epidural analgesia: Outstanding success and disappointing failures. Anaesthesia.

[10] Wu C.F., Hsieh M.J., Liu H.P., Gonzalez-Rivas D., Liu Y.H., Wu Y.C., Chao Y.K., Wu C.Y. (2016). Management of post-operative pain by placement of an intraoperative intercostal catheter after single port video-assisted thoracoscopic surgery: A propensity-score matched study. J. Thorac. Dis..

[11] Ghee C.D., Fortes D.L., Liu C., Khandhar S.J. (2018). A randomized controlled trial of continuous subpleural bupivacaine after thoracoscopic surgery. Semin. Thorac. Cardiovasc. Surg..

[12] Bendixen M., Jørgensen O.D., Kronborg C., Andersen C., Licht P.B. (2016). Postoperative pain and quality of life after lobectomy via video-assisted thoracoscopic surgery or anterolateral thoracotomy for early stage lung cancer: A randomised controlled trial. Lancet Oncol..

[13] Martin L.W., Sarosiek B.M., Harrison M.A., Hedrick T., Isbell J.M., Krupnick A.S., Lau C.L., Mehaffey J.H., Thiele R.H., Walters D.M. (2018). Implementing a thoracic enhanced recovery program: Lessons learned in the first year. Ann. Thorac. Surg..

[14] Fletcher D., Stamer U.M., Pogatzki-Zahn E., Zaslansky R., Tanase N.V., Perruchoud C., Kranke P., Komann M., Lehman T., Meissner W. (2015). Chronic postsurgical pain in Europe: An observational study. Eur. J. Anaesthesiol..

[15] Guimaraes-Pereira L., Valdoleiros I., Reis P., Abelha F. (2016). Evaluating persistent postoperative pain in one tertiary hospital: Incidence, quality of life, associated factors, and treatment. Anesth. Pain Med..

[16] Hedegaard H., Miniño A.M., Warner M. Drug Overdose Deaths in the United States, 1999–2017: NCHS Data Brief: National Center for Health Statistics 2018. https://www.cdc.gov/nchs/data/databriefs/db329-h.pdf.

[17] Singh G.K., Kim I.E., Girmay M., Perry C., Daus G.P., Vedamuthu I.P., De Los Reyes A.A., Ramey C.T., Martin E.K., Allender M. (2019). Opioid epidemic in the United States: Empirical trends, and a literature review of social determinants and epidemiological, pain management, and treatment patterns. Int. J. MCH AIDS.

[18] Hansen H.J., Petersen R.H., Christensen M. (2011). Video-assisted thoracoscopic surgery (VATS) lobectomy using a standardized anterior approach. Surg. Endosc..

[19] Serlin R.C., Mendoza T.R., Nakamura Y., Edwards K.R., Cleeland C.S. (1995). When is cancer pain mild, moderate or severe? Grading pain severity by its interference with function. Pain.

[20] Dindo D., Demartines N., Clavien P.A. (2004). Classification of surgical complications: A new proposal with evaluation in a cohort of 6336 patients and results of a survey. Ann. Surg..

[21] Kehlet H., Jensen T.S., Woolf C.J. (2006). Persistent postsurgical pain: Risk factors and prevention. Lancet.

[22] McKenna R.J. (2008). Complications and learning curves for video-assisted thoracic surgery lobectomy. Thorac. Surg. Clin..

[23] Ziarnik E., Grogan E.L. (2015). Postlobectomy early complications. Thorac. Surg. Clin..

[24] Karcz M., Papadakos P.J. (2013). Respiratory complications in the postanesthesia care unit: A review of pathophysiological mechanisms. Can. J. Respir. Ther..

[25] Kamiyoshihara M., Nagashima T., Ibe T., Atsumi J., Shimizu K., Takeyoshi I. (2010). Is epidural analgesia necessary after video-assisted thoracoscopic lobectomy?. Asian Cardiovasc. Thorac. Ann..

[26] Wildgaard K., Petersen R.H., Hansen H.J., Møller-Sørensen H., Ringsted T.K., Kehlet H. (2012). Multimodal analgesic treatment in video-assisted thoracic surgery lobectomy using an intraoperative intercostal catheter. Eur. J. Cardiothorac. Surg..

[27] Chou R., Gordon D.B., de Leon-Casasola O.A., Rosenberg J.M., Bickler S., Brennan T., Carter T., Cassidy C.L., Chittenden E.H., Degenhardt E. (2016). Management of postoperative pain: A clinical practice guideline from the American Pain Society, the American Society of Regional Anesthesia and Pain Medicine, and the American Society of Anesthesiologists’ Committee on Regional Anesthesia, Executive Committee, and Administrative Council. J. Pain.

[28] Thompson C., French D.G., Costache I. (2018). Pain management within an enhanced recovery program after thoracic surgery. J. Thorac. Dis..

[29] Luketich J.D., Land S.R., Sullivan E.A., Alvelo-Rivera M., Ward J., Buenaventura P.O., Landreneau R.J., Hart L.A., Fernando H.C. (2005). Thoracic epidural versus intercostal nerve catheter plus patient-controlled analgesia: A randomized study. Ann. Thorac. Surg..

[30] De Koning H.J., van der Aalst C.M., de Jong P.A., Scholten E.T., Nackaerts K., Heuvelmans M.A., Lammers J.W., Weenink C., Yousaf-Khan U., Horeweg N. (2020). Reduced lung-cancer mortality with volume CT screening in a randomized trial. N. Engl. J. Med..

[31] The National Lung Screening Trial Research Team (2011). Reduced lung-cancer mortality with low-dose computed tomographic screening. N. Engl. J. Med..

